# Coupling the Structural and Functional Assembly of Synaptic Release Sites

**DOI:** 10.3389/fnana.2018.00081

**Published:** 2018-10-16

**Authors:** Tina Ghelani, Stephan J. Sigrist

**Affiliations:** ^1^Faculty of Biology, Chemistry, Pharmacy, Freie Universität Berlin, Berlin, Germany; ^2^NeuroCure Cluster of Excellence, Charité Universitätsmedizin Berlin, Berlin, Germany

**Keywords:** coupling distances, calcium channel positioning, release sites, active zone assembly, AZ scaffold protein superfamilies

## Abstract

Information processing in our brains depends on the exact timing of calcium (Ca^2+^)-activated exocytosis of synaptic vesicles (SVs) from unique release sites embedded within the presynaptic active zones (AZs). While AZ scaffolding proteins obviously provide an efficient environment for release site function, the molecular design creating such release sites had remained unknown for a long time. Recent advances in visualizing the ultrastructure and topology of presynaptic protein architectures have started to elucidate how scaffold proteins establish “nanodomains” that connect voltage-gated Ca^2+^ channels (VGCCs) physically and functionally with release-ready SVs. Scaffold proteins here seem to operate as “molecular rulers or spacers,” regulating SV-VGCC physical distances within tens of nanometers and, thus, influence the probability and plasticity of SV release. A number of recent studies at *Drosophila* and mammalian synapses show that the stable positioning of discrete clusters of obligate release factor (M)Unc13 defines the position of SV release sites, and the differential expression of (M)Unc13 isoforms at synapses can regulate SV-VGCC coupling. We here review the organization of matured AZ scaffolds concerning their intrinsic organization and role for release site formation. Moreover, we also discuss insights into the developmental sequence of AZ assembly, which often entails a tightening between VGCCs and SV release sites. The findings discussed here are retrieved from vertebrate and invertebrate preparations and include a spectrum of methods ranging from cell biology, super-resolution light and electron microscopy to biophysical and electrophysiological analysis. Our understanding of how the structural and functional organization of presynaptic AZs are coupled has matured, as these processes are crucial for the understanding of synapse maturation and plasticity, and, thus, accurate information transfer and storage at chemical synapses.

## Introduction

Information processing in neural systems relies on accurate and modifiable communication between neurons at chemical synapses. The arrival of an action potential at chemical synapses elicits a Ca^2+^ influx through VGCCs at the presynaptic AZ. A fast and local increase of intracellular Ca^2+^ occurs within the presynaptic bouton and is sensed and translated into a SV fusion event by the SV release machinery. A collection of protein groups constitute the actual SV release machinery, namely SNAREs, (M)Unc13s, (M)Unc18s, complexins, and synaptotagmins, that work in concert to trigger the process of NT release from SVs (Jahn and Fasshauer, 2012).

Synaptic release occurs at defined sites on the presynaptic membrane within an AZ. A multitude of morphological “AZ designs” exist across synapse types and species that, however, are all morphologically characterized by the presence of an electron-dense projection, VGCCs, and SVs at the presynaptic terminus, which together facilitate NT release. Release sites are elaborately assembled by a conserved set of proteins specialized to convey the speed and precision of synaptic transmission (Zhai and Bellen, 2004). Synapses themselves are highly diverse and are grouped based on their differences in response to synapse response time (fast and slow synapses), signal strength, and ability to adapt to signal trains (Gjorgjieva et al., 2016; Jackman and Regehr, 2017). Obviously, variations in the AZ protein composition have evolved to adapt to the functional output of AZs and the synaptic properties on short- and long time scales (Gundelfinger and Fejtova, 2012; Ehmann et al., 2014; Ackermann et al., 2015; Wichmann and Moser, 2015; Van Vactor and Sigrist, 2017). Consequently, synapses can exhibit both high and low transmission fidelity. Work from the past few years has embraced the notion that functional synaptic modulation is essential for directing and programming sensory information within neuronal networks in the brain (Chabrol et al., 2015; Jackman and Regehr, 2017). A number of factors influence the activity-driven functional propensity of synapses to weaken and/or strengthen over time, a spectrum of features collectively referred to as synaptic plasticity. These include factors that may modulate the pre- and postsynaptic sites such as the number, density and location of SVs, VGCCs, postsynaptic receptors, and proteins of the SV fusion machinery (O’Rourke et al., 2012). Topological relations between these molecular players at the nanometer (“nanoscale”) level are now believed to be crucial in defining synaptic efficacy and plasticity and will be discussed in detail below.

Short-term synaptic plasticity refers to forms of rapid and activity-dependent modulations of the efficacy of synaptic transmission, allowing preferred signaling frequencies to behave as temporal filters for synaptic transmission (Hennig, 2013; Jackman and Regehr, 2017). Biophysical studies have come to the consensus that SV release probability and, ultimately, short-term plasticity are fundamentally influenced by the physically coupled distances between VGCCs, and the SVs docked at the presynaptic membrane (Regehr, 2012; Vyleta and Jonas, 2014). During synaptic transmission, SVs sense a VGCC-mediated Ca^2+^ influx through their Ca^2+^ sensor, synaptotagmin, which, in turn, triggers SV fusion in the vicinity of the VGCCs, thus delimiting the SV-VGCC coupling distances at the presynaptic membrane (Wadel et al., 2007; Eggermann et al., 2012; Vyleta and Jonas, 2014; Wang et al., 2016a). Tight coupling occurs within the “nanodomain range” at synaptic sites in which VGCCs and SVs, with their sensors, can apparently be as close as only 10 – 20 nm and well below 100 nm. By contrast, synaptic sites that exhibit loose coupling have their VGCC and SV sensors located farther apart, in the “microdomain range” that spans a region larger than 100 nm. Tight coupling has been associated with synapses that present high release probabilities but here, due to the high release probability, trains of action potentials tend to deplete the SV pools (depressing synapses). By contrast, loose coupling is associated with low release probabilities but typically associated with high-frequency facilitation (Eggermann et al., 2012; Vyleta and Jonas, 2014). Effective coupling distances, with their influence on short-term plasticity, vary across synapse types to possibly provide functional diversity over the brain (Eggermann et al., 2012; Fulterer et al., 2018).

While the molecular mechanisms and proteins controlling these “coupling distances” had remained rather enigmatic for a long time, a few conserved families of AZ proteins have now come forward as regulators of this process. We will revisit, here, the evidence suggesting that these AZ proteins not only mediate tenacity to the ultrastructure of presynaptic AZs but also seem to define stable sites of SV release at the presynaptic membrane, henceforth referred to as release sites. Questions of how such molecular architectures might establish support to SV release sites and how they come to be tightly connected to each other during development are only recently being answered.

The accessibility of *Drosophila* NMJ synapses to a spectrum of *in vivo* approaches, from physiology and intravital imaging to genetics, has made the NMJ a useful platform to study synapses and their AZ initial assembly and subsequent maturation. Here, we review the molecular architecture and organization of *Drosophila* AZs and show how these molecular scaffolds instruct the location of release sites. While placing particular emphasis on the NMJ synapse, we will consider how mechanisms identified at the NMJ synapse may relate or contrast to the operation of other invertebrate or mammalian synapses, toward providing a current and comprehensive understanding of how release sites form and might be regulated.

## Defined Stable Release Sites: an Emerging Concept

Chemical synaptic transmission occurs upon the fusion of SVs at the presynaptic plasma membrane to release the NT stored within. This concept was established by the work done between the 1950s and 1970s, where electrophysiological studies addressing the release of NTs were combined with ultrastructural observations that emerged following the advent of EM. A series of studies by Katz and colleagues on motor neuron NMJ synapses of the frog demonstrated that the release of NTs occurs as a discrete packet (“quantal unit”) rather than as a continuous event (Fatt and Katz, 1952; Del Castillo and Engbaek, 1954; Katz, 1971). These observations were coupled to the first ultrastructural visualizations of synapses. Here, electron-dense regions at the pre- and postsynaptic membrane were observed, pointing toward the presence of proteinaceous specializations. Moreover, the presence of uniformly sized, membrane-bound vesicular structures representing SVs provided the morphological equivalent of the physiologically demonstrated quantal units (De Robertis and Bennett, 1955; Rebhun, 1956; Heuser et al., 1979). Today, SV fusion-mediated chemical transmission is the established synaptic transmission concept that has informed the advances in understanding the functions and organization of presynaptic AZ proteins and the regulation of SV fusion in close proximity to VGCCs.

The sites at which SVs might dock and fuse have been named “release sites” (Pulido and Marty, 2017). The initial evidence for the presence of localized sites of release at the presynaptic membrane was provided through recordings of miniature currents studied under the application of hyperosmotic solutions in hippocampal cultures. Overlapping localizations of these mini currents occur at sites of presynaptic activity, marked by presynaptic proteins and fluorescent-molecule dye uptake localizations (Bekkers et al., 1990; Liu and Tsien, 1995; Auger and Marty, 2000). A series of concerted actions between SVs, their sensors and SNARE proteins occur at such release sites to promote effective release in response to an arriving action potential. Here, although the presynaptic terminal hosts many SVs, only a small fraction of the SVs available have the status of being release-ready. This status is acquired as a consequence of the SV’s positioning and molecular state. The maximum number of vesicles that can be released per action potential usually determines the number of functionally active release sites and is identical to the pool of release-ready vesicles (Neher, 2015; Delvendahl and Hallermann, 2016; Kaeser and Regehr, 2017).

Theoretical calculations of electrophysiological data suggest that maintaining effective transmission and plasticity at a presynaptic terminal should entail a precise arrangement of docked SVs within a nanometer-range distance directly in the vicinity of an activated VGCC. Upon the arrival of an action potential, the VGCCs are triggered open, causing a rapid and steep influx of Ca^2+^ into the presynaptic terminal, which is sensed by the SV release machinery that induces the fusion of primed SVs (Meinrenken et al., 2002; Keller et al., 2015; Nakamura et al., 2015). The Ca^2+^ sensors of the synaptotagmin family, notably synaptotagmin-1 and synaptotagmin-2, promote the fast exocytosis of SVs. EM shows only a small fraction of SVs in the docked state, i.e., in direct contact with the presynaptic plasma membrane. Therefore, only these few SVs possess the fusion competence required for NT release. In order to ensure immediate NT release from an SV, the latter needs to possess the physiological criteria of being a readily releasable vesicle and should be physically docked. After docking but before fusion, SVs might get “primed” through additional biochemical steps (Verhage and Sørensen, 2008; Kaeser and Regehr, 2017). In fact, EM studies of cultured hippocampal synapses show that SVs ultrastructurally docked to the presynaptic membrane might plausibly correspond to the RRP. Here, the processes of SV docking and priming occur simultaneously, and are thus, seemingly the morphological and functional aspect of the same underlying process (Imig et al., 2014). The physical mechanism of SV fusion in itself occurs as a series of well-coordinated reactions that require the concerted action of SNAREs in conjunction with two associate families of priming factors, (M)Unc13 and (M)Unc18 proteins, which are typically essential for the SV release process (Jahn and Fasshauer, 2012; Rizo and Südhof, 2012). A thermodynamically stable, tetrahelical, coiled-coil SNARE complex forms between the SNARE motives of the vesicular protein, Synaptobrevin-2 or VAMP2, and the plasma membrane proteins Syntaxin-1A and SNAP25. This complex provides the energy necessary for the fusion of the SV to the presynaptic membrane by coordinating the binding of the vesicular SNAREs, i.e., VAMP2/Synaptobrevin-2, on the SV, to the target SNAREs Syntaxin-1 (in its open conformation) and SNAP-25 on the plasma membrane. The SNARE complex, thus, facilitates the fusion of the SV membrane into the presynaptic membrane (Jahn and Fasshauer, 2012). Recent studies suggest that the opening of Syntaxin-1 is catalyzed by (M)Unc13, establishing a direct link from the proteins of AZ scaffold to the release machinery at the AZ (Varoqueaux et al., 2002; Ma et al., 2011; Lipstein et al., 2013; Imig et al., 2014; Man et al., 2015).

The detailed structural constituents of release sites and the localization of these proteins within the release sites have only recently come to light. One such recent study shows that the number of the VGCC clusters scales with synaptic size and parallels the number of functional docking sites present at any given AZ. Thus, VGCCs clusters might be present in the close vicinity to each AZ release site (Miki et al., 2017).

Research from several model systems suggests that the number of release sites available limits synaptic transmission and points to a general molecular layout for the largely conserved organization of SV release sites, which, in turn, acts as the fundamental means to control synaptic transmission (Scheuss and Neher, 2001; Müller et al., 2012; Miki et al., 2016). There appear to be only a few defined, active release sites in individual AZs of *Drosophila* NMJs, vertebrate NMJs, and cerebellar mossy fiber bouton synapses (Neher, 2010; Miki et al., 2016). These synapses seem to possess a preferred subset of release sites, which are repetitively reused, during high-frequency transmission, once primed SVs are replenished. This is supported by a recycling pool of SVs with high vesicular release probabilities which promote the rapid recruitment and fusion of SVs to these release sites (Saviane and Silver, 2006; Gaffield et al., 2009; Melom et al., 2013; Peled et al., 2014; Ritzau-Jost et al., 2014). The Calyx of Held, another important model synapse, employs many release sites in a parallel fashion, with each release site displaying a rather low probability of SV release. The parallel release from these many sites generates high synaptic transmission rates which allow sustained stimulation at these synapses (Neher and Sakaba, 2008; Borst and Soria van Hoeve, 2012). Notably, differentiation in release site functionality within a single AZ may also occur, where release sites at the periphery of an AZ are expected to be prone to low-release probabilities and have a lower frequency of fusion events, while a higher frequency of fusion is expected at central locations of release sites (Böhme et al., 2016; Reddy-Alla et al., 2017; Byczkowicz et al., 2018).

In this review, we will delve into the details of the localization, composition, and formation of release sites that mediate both low and high frequency transmission at synapses.

## Ultrastructural Organization of Active Zones and Release Sites

Presynaptic terminals possess proteinaceous assemblages that form a dense, insoluble “cytomatrix” of AZ proteins (“cytomatrix of the AZ”), which in EM is reflected as electron-dense projections that extend from the AZ plasma membrane into the cytoplasm. Presynaptic AZs organize the SV release machinery by performing four key functions: sequestering SVs to release sites, priming SVs for rapid Ca^2+^-triggered fusion, localizing VGCCs adjacent to SV release sites, and coordinating trans-synaptic signaling for precise alignment of pre- and postsynaptic elements (Südhof, 2012).

In an effort to understand the ultrastructural principles of release site definition, studies employing EM and/or super-resolution light microscopy at AZ sites have, over the years, given us a clearer picture of how the AZ building blocks, namely VGCCs, SVs and AZ proteins, interact to build release sites. In addition, these imaging studies have delineated a spectrum of AZ morphologies or architectures, henceforth referred to as AZ designs.

Comparing the ultrastructural design of different AZ types allows for a division of AZs into morphologically distinct groups, i.e., those with elaborate electron-dense projections, such as T-bars and ribbons, and those with less prominent dense projections, such as those of *C. elegans* and AZs present at most vertebrate CNS synapses. Vertebrate and invertebrate sensory synapses and NMJ synapses generally have prominent and well-defined dense bodies, while the ultrastructural elements of vertebrate CNS synapses often appear less prominent in EM (Ackermann et al., 2015).

Early electron micrographs of AZs of vertebrate NMJs revealed dense elongated structures at the presynaptic membrane, constituting double rows of proteins that possibly contain VGCCs and are surrounded by SVs (Couteaux and Pécot-Dechavassine, 1970; Heuser et al., 1979). Consistent with this observation, the ultrastructural “design” at frog NMJ AZs has two rows of SVs that form in parallel toward the outside of an AZ density and likely include their VGCCs (Harlow et al., 2001). Electron micrographs of *C. elegans* NMJs reveal AZs that have a broad surface of plasma membrane with electron-dense projections at both pre- and postsynaptic sites (Zhai and Bellen, 2004; Stigloher et al., 2011; Ackermann et al., 2015). Vertebrate synapses show related AZ designs. Tomograms of hair cell ribbon synapses and electron micrographs of rodent NMJs exhibit SVs localized between two rows of VGCCs and the AZ density, forming a central station for SVs to dock (Frank et al., 2010). Meanwhile, AZs of vertebrate central synapses are, by and large, less elaborate than AZs of sensory synapses but exhibit fine filamentous projections or “tethers” that connect SVs, located up to 100 nm from the plasma membrane, thus, obviously “holding them” close to release sites (Siksou et al., 2007; Zhou et al., 2013; Midorikawa et al., 2014; Ackermann et al., 2015). At *Drosophila* NMJs, AZs form a platform consisting of a meshwork of filaments overlaying a “pedestal,” aptly known as “a T-bar.” The filamentous protrusions span approximately 100 – 200 nm from the AZ membrane into the cytoplasm, and have VGCCs centered directly under the T-bar “pedestal” and SVs accumulating on the top roof of the T-bar (Jiao et al., 2010; Wichmann and Sigrist, 2010). Similar T-bar-like architectures are generally found at the AZs of both peripheral (such as NMJs) and most central synapses of all insects (Zhai and Bellen, 2004; Hamanaka and Meinertzhagen, 2010; Fulterer et al., 2018). Regardless of the seemingly complicated design of AZs, their core ultrastructure harbors a dense and proteinaceous cytomatrix of AZ material that links the SVs, VGCCs and the presynaptic plasma membrane together. For a detailed description and comparison of the different AZ morphologies in various species and synapses, please refer to the following: Zhai and Bellen (2004), Ackermann et al. (2015), and Slater (2015).

All these different AZ designs probably possess a common concept for the ultrastructural organization at the presynaptic terminus, as they all possess an electron-dense projection at the presynaptic membrane, which contains the AZ protein cytomatrix, SVs that are tethered around these dense projections, and VGCCs that are positioned in the presynaptic membrane in close proximity to SVs. Despite the apparent structural heterogeneity between the AZ ultrastructure of all these various synapse types, their presynaptic AZ sites support the docking of SVs at defined release sites on the presynaptic membrane. During this process, the AZ scaffold takes up various roles in the recruitment and consequent delivery of SVs to the presynaptic membrane. Therefore, a simple and common concept of building the presynaptic AZ ultrastructure also likely instructs the canonical design of release sites and helps define the lateral distances between SVs and VGCCs at these sites.

### A Confined and Conserved Set of Extended Proteins for Forming Active Zones

Recent studies have arrived at a consensus that a defined “set” of scaffold proteins, largely conserved between vertebrates and invertebrates and across different synapse types, form an AZ scaffold at the presynaptic terminus (Owald and Sigrist, 2009; Südhof, 2012; Ackermann et al., 2015). A conserved protein constituency between synapses points to the presence of basic building blocks of AZ proteins that may supply all AZ sites and suggest that these proteins are likely at the core of an AZ’s principal functionality; based off ancient conserved principles.

These conserved protein complexes entail the following conserved components: the ELKS/CAST/BRP family, the large RIM family (that also includes its more distant members, such as the mammalian Piccolo and Bassoon, as well as *Drosophila* Fife), the RIM-BP family, the (M)Unc13 family, the Liprin-α/Syd-2 and, finally, the Syd-1 family members (Südhof, 2012; Gundelfinger et al., 2016; Petzoldt et al., 2016; Xuan et al., 2017). Together, these AZ proteins are thought to be essential for synapse tenacity, localization of SVs to their fusion site and positioning of VGCCs.

Proteins within each of these families possess a significant degree of similarity in the fundamental design of their molecular domain structure, which probably allows for a relatively conserved function of these proteins across synapse variations. In this section, we have, therefore, grouped these AZ proteins into structurally related superfamilies of proteins.

**FIGURE 1:**
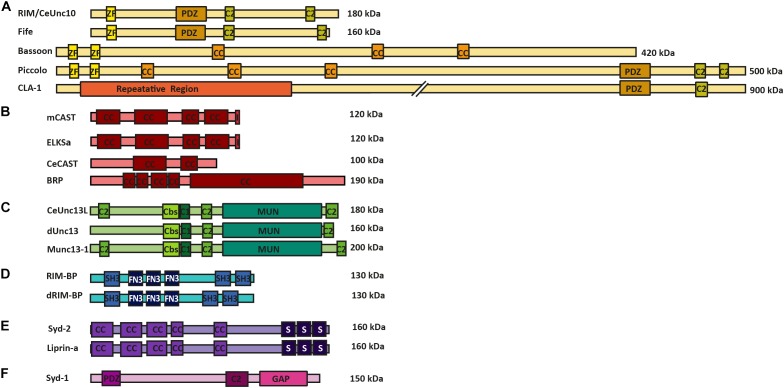
AZ protein superfamilies in invertebrates and vertebrates. Molecular structure of AZ proteins grouped into superfamilies. **(A)** RIM superfamily is composed of RIM/Unc10, Bassoon, Piccolo, Fife, and Clarinet. These proteins possess an N-terminal zinc-finger domain (ZF), a PDZ domain and two C2 domains. Mammalian Bassoon and Piccolo proteins additionally possess three coiled-coil domains (CC). **(B)** ELKS/CAST/BRP superfamily of proteins contains multiple CC regions and an additional C-terminal IWA motif in the mammalian isoforms (I). **(C)** M(U)nc13 superfamily consists of Unc13L (*C. elegans*), Unc13 (**A,B** isoforms in *Drosophila*) and Munc13-1—4 in mammals. These proteins have two/three C2 domains, a calmodulin binding site (Cbs) (with the exception of Unc13B), a C1 domain, and a MUN domain. **(D)** dRIM-BP (*Drosophila*) and RIM-BP form a superfamily of AZ proteins that possess an interruption of three contiguous FN3 domains between their first and last SH3 domains **(E)** Syd-2 /Liprinα family contain five CC regions and three C-terminally located SAM domains. **(F)** Syd-1 family possess a PDZ domain, C2 domain and a unique Rho-GAP domain.

Potentially, the most diversified superfamily of AZ proteins is the **RIM (Rab3-interacting molecule) superfamily**. This family includes the standard RIM isoforms and Fife (in *Drosophila*), the recently found CLA-1 (in *C. elegans*), and the large scaffolding proteins Bassoon, Piccolo and Piccolino (in mammals). These proteins function by providing an interaction surface for a number of AZ proteins, while binding directly to Munc13s and RIM-BPs, thus, promoting close interaction of the linked AZ proteins to the SV release machinery (Dulubova et al., 2005). In addition, these proteins are also involved in VGCC recruitment and clustering, thus, providing AZ proteins involved in SV release in close proximity to the site of release induction. These interactions are achieved via the PDZ domains, C2 domains and Zinc finger motifs that all these proteins possess in common with each other (**Figure 1A**) (Dick et al., 2003; Dulubova et al., 2005; Khimich et al., 2005; Lu et al., 2006; Frank et al., 2010; Südhof, 2012; Alvarez-Baron et al., 2013; Xuan et al., 2017).

The **CAST** and Glutamic acid (**E**), leucine (**L**), lysine (**K**) and serine (**S**)-rich protein **(ELKS) proteins** (at mammalian synapses), **CeCAST** (at *C. elegans* synapses) and **BRP** (at *Drosophila* synapses) superfamily of proteins are characteristic for their continuous coiled-coil architecture. Mammalian ELKS protein is widely expressed over a number of tissue types and has numerous splice variants (Nakata et al., 2002). The more specific expression of CAST protein and ELKSα isoform is brain-specific and present at the AZs of these synapses (Ohtsuka et al., 2002; Deguchi-Tawarada et al., 2004). In addition, these proteins share up to 70% protein homology with one another and, due to this similarity, are proposed to have related but discrete functions within a synapse, as they reportedly localize at different positions within the AZ site (Ohtsuka et al., 2002; Deguchi-Tawarada et al., 2006). For a comprehensive overview of the diverse functional roles of synaptic and non-synaptic ELKS that bolster the protein’s role as an intrinsic scaffold protein and an organizer of SV traffic please see the review of Kaeser and Held (2018). One representative protein member each from this superfamily is found in the *C. elegans* and the *Drosophila* model systems, namely CeCAST and BRP, respectively (**Figure 1B**) (Kittel et al., 2006; Wagh et al., 2006). Proteins of this superfamily all possess four or five conserved coiled-coil regions. However, the mammalian CAST/ELKS members possess a C-terminal IWA motif, which is required for binding to RIM1’s PDZ domain, that is not present in their invertebrate counterparts. On the other hand, at *Drosophila* synapses, the N-terminal half of BRP resembles the N-terminal region of mammalian ELKS/CAST, but it also harbors an extended C-terminal region, only present in insects, that forms the T-bar pedestal present at their AZs (Hida and Ohtsuka, 2010).

The third superfamily of proteins is that of **(M)Unc13** proteins which connects the scaffold proteins to the actual SV fusion machinery. (M)Unc13 superfamily members possess a general domain structure that includes at least one MUN domain, a C1 domain, a C2 domain and often a Ca^2+^-calmodulin and diacylglycerol-binding site. This general domain design comes with an exception for Munc13-4/Unc13D, which lack the Ca^2+^-calmodulin and diacylglycerol binding sites (Koch et al., 2000; Crozat et al., 2007; Dudenhöffer-Pfeifer et al., 2013). The MUN domain is used in the protein family’s central function of SV priming and release by enabling the transition from the Munc18-1 closed syntaxin-1 complex into the SNARE complex (Richmond et al., 2001; Basu et al., 2005; Ma et al., 2011). In addition, many isoforms of this protein family display a Ca^2+^-calmodulin binding site close to their C1 and C2 domains. The C2 domain itself binds Ca^2+^ and phosphatidylinositol phosphates, which, in turn, potentiate release probability (Shin et al., 2010) (**Figure 1C**), while the C1 domain is involved in diacylglycerol phorbol ester-dependent regulation of SV release (Rhee et al., 2002; Basu et al., 2007) (**Figure 1C**). There are four vertebrate Munc13 members (Munc13-1–4) of which Munc13-1–3 are brain specific, while the *Drosophila* and the *C. elegans* systems have fewer Unc13 members each; namely, Unc13A and B and Unc13L, S and D, respectively (Brumell et al., 1995; Dulubova et al., 2005; Lu et al., 2006; Böhme et al., 2016). These proteins are crucial for promoting SV docking and regulation of SV fusion at the respective synapse types, at which they are found (Dulubova et al., 2005; Lu et al., 2006; Böhme et al., 2016; Reddy-Alla et al., 2017).

The **RIM-BP superfamily** members are important for the structural integrity of the AZ scaffold and probably also play direct roles in organizing protein architectures for NT release. These proteins have three SH3 binding domains in common, interrupted by three contiguous fibronectin-type III domains (**Figure 1D**). The first SH3 domain of the mammalian RIM-BP has been found to interact with AZ protein Bassoon, while the last SH3 domains of RIM-BP and dRIM-BP (*Drosophila* RIM-BP) are involved in the direct interaction to RIM1 and VGCCs (Wang et al., 2000; Hibino et al., 2002; Liu et al., 2011). The RIM-BP’s direct interactions to VGCCs link them to release sites and to RIMs, allowing RIM-BPs to regulate VGCC positioning at the presynaptic AZ site (Hibino et al., 2002; Kaeser et al., 2011).

The **Liprin-α/Syd-2 superfamily** of proteins was originally found as binding partners to tyrosine phosphatase LAR. All Liprin-α isoforms possess a highly similar domain organization (**Figure 1E**), consisting of coiled-coil domains that provide interaction surfaces for binding to several synaptic adapter proteins, including CAST, GIT1, RIM, and KIF1A, to their N-terminal region (Schoch et al., 2002; Ko et al., 2003; Shin et al., 2003). At their C-terminal end three sterile alpha motif (SAM) domains are found, which possess binding sites to both protein phosphatases (e.g., LAR, PTPδ and PTPσ) and protein kinases (e.g., CASK). These regions also promote heterodimerization between Liprin isoforms, (Serra-Pagès et al., 1995). Two families of vertebrate Liprins exist as Liprin-α and Liprin-β, and have these four (α1, α2, α3, and α4) and two (β1 and β2) members, respectively (Serra-Pagès et al., 1995, 1998). In invertebrates, *Drosophila* has only one member of Liprin-α and β each, also known as Liprin-α/β, while the *C. elegans* encode only a single Liprin-α isoform called Syd-2 (Zhen and Jin, 1999; Kaufmann et al., 2002). The Liprin-α/Syd-2 superfamily members have key roles in AZ formation as they bind to a number of AZ superfamily members such as, Syd-1, Unc13, RIM, and ELKS, thus sequestering them to developing AZs (Ohtsuka et al., 2002; Schoch et al., 2002; Wang et al., 2002; Ko et al., 2003; Dai et al., 2006; Patel et al., 2006; Ackermann et al., 2015).

**Synapse defective-1 (Syd-1)** falls into a group of its own, as it is structurally dissimilar to the Liprin-α family. This protein was first discovered in *C. elegans* and has been heavily investigated for its ability to regulate AZ assembly at invertebrate synapses. The Syd-1 protein shares certain structural similarities to the RIM family, in that it possesses a PDZ and C2 domain, with the exception that Syd-1 has its own signature rho-GTPase activating protein domain, which is not shared by any other AZ superfamily. Syd-1 is involved in promoting BRP clustering and AZ assembly at *Drosophila* AZs, and neurite outgrowth in *C. elegans* (Hallam et al., 2002; Owald et al., 2010; Spinner et al., 2017) (**Figure 1F**).

Members from each of these superfamilies are required in concert to coordinate AZ structural assembly as well as AZ functionality, probably to a good degree by providing SV release sites at a defined distance from the VGCCs. The details of these aspects will be a central topic of discussion in the following sections.

### Functional Roles of Active Zone Scaffold Proteins

A body of work has established that AZ scaffold proteins promote SV priming and docking, help confer suitable SV release probability, and are responsible for the localization of VGCC to AZs and release sites (Kaeser et al., 2011; Acuna et al., 2015; Grauel et al., 2016; Reddy-Alla et al., 2017). In an effort to understand their individual role, more than a decade of work employing super-resolution light microscopy and EM, combined with biochemical and electrophysiological analyses on loss-of-function mutants of AZ proteins have revealed the many interaction partners and the partial functional redundancies in the roles of AZ scaffold proteins (Kittel et al., 2006; Kaeser, 2011; Liu et al., 2011; Müller et al., 2012; Liu et al., 2014; Acuna et al., 2015).

A series of structure-function analyses from individual and double-mutant loss-of-function experiments have come a long way in revealing AZ scaffold core functions in SV release, VGCC localization, and coupling at invertebrate synapses. Only one VGCC α1 subunit isoform Cac is expressed at *Drosophila* NMJ synapses, which corresponds to the mammalian CaV2.1/2.2 (N or P/Q type VGCCs) and is responsible for synaptic transmission at these synapses. Null mutants of *cac* are embryonically lethal, probably due to their nearly complete incapability to permit any evoked SV release (Kawasaki et al., 2002; Hou et al., 2008). Intracellular binding partners of Cac, thus, possess the proclivity to influence synaptic transmission. Only a few *Drosophila* AZ scaffold proteins possess such interactions with Cac, of which BRP is a core AZ scaffold member (Fouquet et al., 2009).

BRP is present as both a 170 kDa (short) and a 190 kDa (long) isoform at NMJ synapses. The latter isoform possesses an additional and exclusive N-terminal domain that the BRP-170kDa isoform lacks. At NMJ AZs, the two isoforms form discrete oligomers that alternate in a circular array to form a donut-shaped structure centered at the AZ site, as was visualized by sub-diffraction resolution stimulated emission depletion (STED) fluorescence microscopy (Kittel et al., 2006; Matkovic et al., 2013). The BRP protein forms an intrinsic component of the synapse’s prominent electron-dense T-bar structure and shapes it directly (Wagh et al., 2006; Fouquet et al., 2009). Subsequent work employing super-resolution microscopy approaches such as; STED applications and direct stochastic optical reconstruction microscopy, have revealed the precise nanoscopic localization of BRP within the AZ, and revealed that BRP signals when resolved appear as polarized filamentous proteins that possess an elongated and oriented structure, wherein their N-termini face the presynaptic membrane, while their C-termini extend into the bouton’s cytoplasm (Fouquet et al., 2009). In addition, these studies show that the nanoscopic organization of BRP, within an AZ, can demarcate the different physiological states the AZ undergoes and along with the resolved localizations of other AZ components, such as VGCCs, RBP, Syd-1 and Liprin-α, have begun to provide an increasingly detailed picture of the AZ scaffold at *Drosophila* NMJs (Fouquet et al., 2009; Owald et al., 2010; Liu et al., 2011; Maglione and Sigrist, 2013; Ehmann et al., 2014).

Previous studies of the NMJ synapse have shown clustering of Cac at presynaptic AZ membranes (Kawasaki, 2004). This Cac clustering becomes inefficient at BRP mutant AZs, which also display a complete loss of their T-bar structures and inefficiently evoked SV release observed in response to discrete action potentials. However, *brp* null mutants showed an atypical facilitation in response to higher frequencies of action potentials (Kittel et al., 2006). This changed short-term plasticity suggests an increased distance between Cac and release-ready SVs. Consistently, an increased sensitivity versus slow Ca^2+^ chelator EGTA-AM was observed at these mutant synapses, suggesting an altered coupling, from tight to loose, between VGCCs and SV-Ca^2+^ sensors and, thus, revealing a fundamental role for BRP in VGCC localization (Kittel et al., 2006). This relationship was further established in a later study where the N-terminal region of the BRP, in its 190 KD isoform, was found to bind directly to the intracellular C-terminal region of Cac and solidified BRP’s function in organizing the VGCC’s nanodomains at the presynaptic AZ site (Fouquet et al., 2009).

While the N-terminal region of BRP is conserved and similar to other members of its superfamily, BRP’s C-terminus seemingly plays a significant role in SV recruitment, unlike its mammalian superfamily members (Hallermann et al., 2010). A study at NMJ synapses describing a hypomorph allele of *brp* (“*brp-nude*”), which is devoid of only the very last 1% of its C-terminal sequence, revealed properly formed T-bar structures atop undisturbed VGCC localizations at the presynaptic membrane. These T-bar structures, however, lack the normal and expected accumulation of SVs atop the T-bar distal aspect (or “roof”).

Electrophysiological recordings at these mutant synapses showed unaltered basal SV release, while paired-pulse protocols provoked an atypical depression and sustained stimulations caused an atypically slow recovery (Hallermann et al., 2010). These results link the AZ scaffold’s ability to tether SVs directly with SV recruitment. The BRP protein possibly engages in physically moving these SVs to precise localizations on the presynaptic membrane and into release slots, thus, facilitating efficient sustained release at an AZ site.

In contrast to the observations of the *brp* null mutants, the isoform-specific BRP mutants (lacking either the 190 or the 170 kDa isoform) did not show major Cac clustering or SV release probability deficits although the basal synaptic transmission was reduced, as a result of the reduced numbers of SVs in the RRPs. Consistently, the number of docked SVs were also significantly reduced, suggesting the BRP architecture at the AZ site may be involved in regulating the RRP size by influencing the number of VGCC-coupled SV release sites (Matkovic et al., 2013). Extended studies on isoform-specific BRP mutants have also shown that the N-terminal Cac-binding region of BRP, unique only to its 190 kDa isoform, is not sufficient for a complete declustering of VGCCs. This would suggest an involvement of multiple AZ scaffold proteins that probably employ a collective mechanism to anchor VGCCs to the presynaptic membrane (Matkovic et al., 2013). This may certainly be a mechanism employed at the AZ site, since the RIM-BP superfamily members also bind VGCCs at rodent and *Drosophila* AZs directly, and display a partial loss of VGCCs at *drim-bp* null NMJs (Wang et al., 2000; Hibino et al., 2002; Matkovic et al., 2013). In addition, STED microscopy of dRIM-BP at NMJ *Drosophila* synapses shows that the protein is localized to the AZ core, overlaying the central AZ localizations of Cac, thus, physically positioning dRIM-BP as a prime candidate for nanodomain coupling at AZ sites (Liu et al., 2011).

A series of studies at *Drosophila* NMJ synapses involving *drim-bp* null mutants and a dRIM-BP hypomorph (with 30% residual expression) uncovered a dramatic effect on AZ scaffold ultrastructure and synaptic transmission. The *drim-bp* null mutants displayed 90% reduction in baseline transmission, while the frequency and amplitude of their miniature excitatory junctional currents remained unchanged, causing a dramatic reduction in the number of SVs released per action potential in these mutants. In addition, a 10 Hz paired-pulse stimulation assay on *drim-bp^stop1^* null mutant AZs was met with a strong facilitation, although a consecutive five-pulse, 100 Hz stimulation was met with a sizable recovery that matched control levels. Taken together, this work demonstrates that SV release probability in dRIM-BP mutants is severely affected, although the SV release itself may be raised back to rather normal levels under high-frequency stimulation, arguably implying that dRIM-BP does not influence the SV fusion process or release machinery but rather an upstream component (Liu et al., 2011). In addition, evoked SV fusion events appeared desynchronized at these synapses as a significantly increased excitatory junctional current rise time was observed. In addition, Cac-GFP signals were consistently reduced by ∼25% over the entire NMJ of all three dRIM-BP null alleles and the hypomorphic allele. The *drim-bp* null mutants also exhibit an altered ultrastructure of the electron-dense material, as the T-bars of these mutant AZs appear severely misshapen (Liu et al., 2011). Overall, loss of *drim-bp* function results in ultrastructural changes and severe impairment of baseline SV transmission, possibly as a result of disturbed SV-VGCC coupling, leading to desynchronized release at the presynaptic membrane (Liu et al., 2011).

In addition, the RIM superfamily of proteins has also been shown to regulate baseline synaptic transmission, cluster VGCCs and facilitate SV priming at mammalian AZs. The RIM function at *Drosophila* NMJ AZs appears evolutionarily conserved, as *rim* null mutants displayed baseline SV release deficits, lowered Ca^2+^ influx and channel number, reduced RRP size and a decrease in AZ numbers, resulting in a severe impairment of evoked SV release at these synapses (Graf et al., 2012; Müller et al., 2012). The *drim-bp* mutations generally display a stronger defect in baseline transmission followed by a dramatic facilitation when compared to the *rim* mutants, although both mutants show consistent defects in SV transmission and Ca^2+^ influx, thus, implicating the presence of a protein complex that works in a coordinated fashion. In addition, although VGCC numbers are reduced in *rim* mutants, the core scaffold protein BRP remains unaffected and tagged RIM expression in *brp* mutants of *Drosophila* and *C. elegans* synapses appears undisturbed at AZ sites. This suggests that BRP is not involved in RIM localization to release sites (Deken et al., 2005; Graf et al., 2012). Furthermore, these results suggest that these AZ scaffold proteins may perform partially overlapping roles here, as the disruption of only one of the RIM, dRIM-BP, or BRP proteins does not completely eliminate VGCC localization at their release sites.

An interesting additional function of the RIM protein in regulating synaptic homeostasis was uncovered in loss-of-function *rim* mutants at NMJ synapses. Homeostatic modulation of presynaptic NT release is an evolutionarily conserved form of plasticity documented at the NMJs of a number of systems, from *Drosophila* to humans, and occurs upon a postsynaptic NT receptor block that elicits an increase in SV release, offsetting the magnitude of receptor inhibition precisely. No homeostatic enhancement of SV release occurs at *Drosophila* NMJs of *rim* null mutants during a postsynaptic receptor inhibition. Two presynaptic processes are critical for synaptic homeostasis. The first is the enhancement of presynaptic Ca^2+^ influx to potentiate SV release, which, despite the initial baseline Ca^2+^ influx defect, can be re-sequestered during homeostasis in *rim* null mutants. The second is the provision of an enhanced RRP size, which is blocked in *rim* null mutants and, hence, a RIM-dependent modulation of the RRP size appears to be essential for the homeostatic plasticity response (Müller et al., 2012). A recent study has shown that the α2δ-3 subunit of VGCCs functions together with RIM, either directly or indirectly, to achieve a homeostatic potentiation of the RRP size and may, in concert with other proteins at the release site, regulate synaptic homeostasis (Wang et al., 2016b).

Taken together, deletions of members of the RIM, ELKS/BRP and RIM-BP superfamilies at *Drosophila* synapses display disrupted AZ scaffold structures. This ultrastructural effect is compounded by impaired SV priming and, subsequently, SV transmission in *rim-bp* mutants, and impaired VGCC clustering in the *brp* mutants. Meanwhile, RIM proteins probably operate downstream of BRP and RIM-BP function, as they potentiate the homeostatic regulation of the RRP size at AZs; this RIM-mediated effect can be perceived in the presence of unaltered BRP-regulated Ca^2+^ influx and RBP-mediated SV priming and SV release (Kittel et al., 2006; Fouquet et al., 2009; Liu et al., 2011).

Furthermore, these results concur with data from vertebrate synapses. Here, RIM and ELKS, through an exhaustive cohort of biochemical studies, have been shown to interact with a number of other AZ scaffold proteins and obviously function by providing a smart scaffold surface for other AZ proteins and potentially the release machinery to bind to. Individual knockout studies of these proteins, however, do not paint a clear picture (Ohtsuka et al., 2002; Schoch et al., 2002; Wang et al., 2002; Takao-Rikitsu et al., 2004). Individually, RIM1, RIM2, ELKS-1 and ELKS2 knockout mice have largely intact AZ scaffolds, with the remote changes observed in SV clustering, levels of Munc13-1 in the RIM knockouts and an increased RIM solubility in the ELKS knockout studies (Schoch et al., 2002; Kaeser et al., 2009; Deng et al., 2011; Liu et al., 2014; Held et al., 2016). Importantly, however, deletion of all RIMs in mice perturbs the localization of VGCCs, as well as SV priming and tethering, while, even here, the gross AZ scaffold architecture remained rather unchanged (Han et al., 2011; Kaeser et al., 2011). Deleting RIM-BP1 and RIM-BP2 proteins at synapses of cultured hippocampal neurons and Calyx of Held synapses had only a mild effect on SV release and no clear structural effect (Acuna et al., 2015). Through these studies, it appears that loss of individual AZ proteins could be largely compensated, again suggesting at least a certain degree of functional “redundancy” between AZ scaffold proteins. This is probably because vertebrates encode several loci and gene products of relevant genes that, in turn, complicate their analysis. Such findings have motivated the simultaneous deletion of representatives from distinct AZ superfamilies to expectantly perturb the AZ scaffold in its entirety. Indeed, quadruple knockouts of RIM1, RIM2, RIM-BP1 and RIM-BP2 proteins in mice exhibit a total loss of NT release from severe impairments in SV priming and docking, a dramatic loss of AZ scaffold density, and a trans-synaptic effect that disrupts the organization of the postsynaptic density (Acuna et al., 2016). This study revealed a redundancy in the role of the two RIM and RIM-BP protein families in maintaining global AZ functions. Similar conclusions were also drawn from work on mice completely devoid of RIM1αβ and RIM2αβγ, together with ELKS1α and ELKS2α isoforms (Wang et al., 2016a). Consequently, cultured hippocampal synapses of these mutant mice lose Munc13, Bassoon, Piccolo and RIM-BP following a mass disassembly of the AZ scaffold (Wang et al., 2016a).

The RIM, RIM-BP and ELKS superfamilies of proteins, at vertebrate and invertebrate synapses, function collectively to structurally and functionally uphold the organization of the AZ scaffold. Within the AZ scaffolds, AZ superfamily members BRP, RIM-BP, and RIMs support VGCC clustering at AZs and through their interaction concentrate VGCCs and the Ca^2+^ influx. This, in turn, would enable regulation of release probability and promote evoked SV release (Wadel et al., 2007; Kittelmann et al., 2013; Ehmann et al., 2015; Van Vactor and Sigrist, 2017). In addition, the AZ superfamily members mentioned have been implicated in the direct maintenance of release sites and VGCC density, while their sheer protein levels can also predict an AZ site’s release probability (Kittel et al., 2006; Wagh et al., 2006; Han et al., 2011; Kaeser et al., 2011; Liu et al., 2011; Acuna et al., 2015). Therefore, taken together, these studies implicate AZ scaffold proteins in localizing VGCCs and release factors to appropriate positions to execute their function in SV release.

### Toward a Molecular Definition of Release Sites

*A priori*, release site-regulating protein should be located at the release site on the presynaptic membrane, and likely becomes stably integrated at this given site. Moreover, these proteins might take a role in SV priming and/or ultrastructural SV docking to the presynaptic membrane.

**FIGURE 2:**
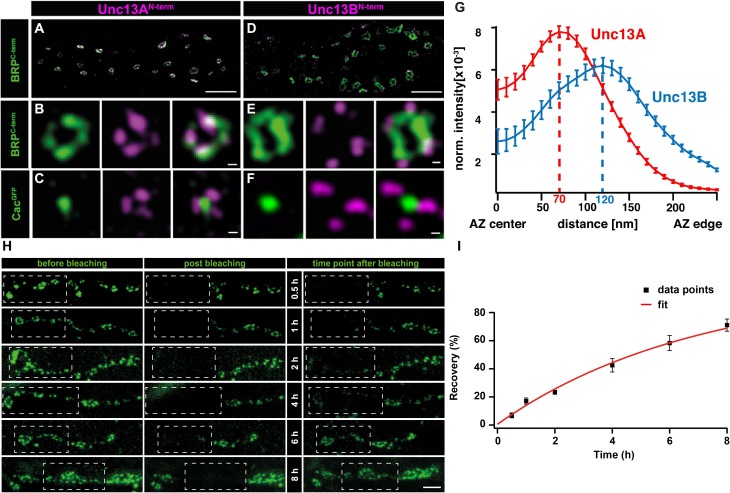
Stable and specific positioning of Unc13A and B at *Drosophila* NMJ synapses. **(A,D)** Two-color STED microscopy images of synaptic boutons or individual planar AZs **(B,C,E,F)** from third instar larvae of the displayed genotypes labeled with antibodies to indicated proteins. **(G)** Mean intensity profile of Unc13A and Unc13B immunoreactivity plotted from the center of the AZ (the reference center being that of the BRP signal). The intensity maximum of the average fluorescence profile was found 70 nm from the AZ center for Unc13A and at 120 nm for Unc13B. Scale bars: **(A,D)**: 1.5 μm; **(B,C,E,F)**:50 nm; **(H)** 250 nm (Modified from Böhme et al., 2016). **(H)** Long-term FRAP of motoneuronally overexpressed Unc13A-GFP at muscle 26/27 depicts a stable integration of Unc13A that requires hours to recover completely. Dashed box shows bleached bouton before and directly after the fluorescence bleaching. Fluorescence recovery was measured 0.5; 1; 2; 4; 6; or 8 h after bleaching. Different time points were measured in different animals. **(I)** Quantification of percentage recovery over time. Bleached Unc13A-GFP showed a slow fluorescence recovery, exhibiting a tau of 6.88 ± 0.55 h, single exponential recovery fit. Data are mean ± SEM. Scale bar:5 μm (Modified from Reddy-Alla et al., 2017). Reproduced with permission, from Böhme et al., 2016 **(A—G)** and from Reddy-Alla et al., 2017 **(H,I)**.

Recent work on *Drosophila* NMJ synapses shows that the M(U)nc13 superfamily member, Unc13A, fulfills all the criteria for a release site-generating molecule (Böhme et al., 2016). STED imaging reveals Unc13A clusters positioned in close proximity to VGCCs at precise localizations within the AZ, i.e., at a 70 nm distance from the centrally located VGCC clusters. A novel assay that employs the postsynaptic expression of fluorescent Ca^2+^-indicators (GCaMPs) allows the recording of events of evoked and spontaneous release at individual NMJ AZs (Melom et al., 2013; Peled et al., 2014; Walter et al., 2014; Muhammad et al., 2015). Applying this assay revealed that presynaptic Unc13A levels could predict the probability of a given AZ to engage in an AP-evoked release. Meanwhile, the ultrastructural data showed Unc13A cluster positions coinciding with the locations of docked SVs. In addition, these Unc13A clusters were found to be highly stable, as intravital live imaging studies, followed by a FRAP assay, reveal only a very slow turnover of this protein, which only occurs over a span of hours, ensuring its stable integration at AZ release site locations (**Figures 2H,I**) (Böhme et al., 2016). Lastly, but most importantly, deleting the scaffold-binding N-terminus of Unc13A changes the localization, position, and efficacy of SV release sites and undermines the scaling of evoked release events and AZ size (Reddy-Alla et al., 2017). All these studies clearly implicate Unc13A as the release site defining protein at *Drosophila* synapses. Interestingly, in *brp–rim-bp*, as well as in *rim–elks* and *rim–rim-bp* double mutants, the synaptic localization of the release site-generating molecules, Unc13A and Munc13-1, at *Drosophila* and vertebrate synapses, was largely impaired, conforming to the loss of release sites observed in these mutants, thus, demonstrating a close-knit relation between the AZ scaffold and release site regulation (Acuna et al., 2015; Böhme et al., 2016; Wang et al., 2016a).

A recent study by Sakamoto et al. (2018) combined glutamate sniffers, which sense glutamate release at high signal to noise ratios, to couple imaging of quantal release events with super-resolution imaging of AZ components. Consistent with the work at *Drosophila* synapses, this elegant analysis showed that Munc13-1 but not SNAREs localize stably to active glutamate release sites (Sakamoto et al., 2018). Local “supramolecular” Munc13-1 assemblies seem to provide a local surface to which open syntaxin-1 molecules can bind, that, in turn, generates activated SNARE complexes to locally promote SV priming and docking at these sites. In other words, this study agrees that stable (M)unc13 clusters form release sites at the presynaptic AZ membrane.

Taken together, it seems that the mechanistic rationale behind an AZ scaffold localizing in the close vicinity of VGCCs clusters and the SV fusion machinery is to provide a stable platform for (M)Unc13 superfamily members to generate and regulate release sites.

### Release Sites and the Control of SV-VGCC Coupling

For a long time, coupling distances, defined as the physical distance from the closest VGCC to the Ca^2+^ sensors, present on SV membranes, were a purely biophysically derived entity and the relevant proteins creating such distances were themselves not visualized. Measuring differential sensitivity of SV release versus exogenous Ca^2+^ buffers, EGTA and BAPTA, that vary in their Ca^2+^ sequestering speeds, was a dominant assay to biophysically measure effective coupling distances at a variety of synapses. These measurements predicted a spread of effective coupling distances, being 10–20 nm at the basket cell-Purkinje cell synapses and basket cell-granule cell synapses, <30 nm at the granule cell-Purkinje cell synapses, 30–60 nm at the Calyx of Held synapses, and ∼65–90 nm at the CA3 pyramidal cell synapses of mossy fibers (Bucurenciu et al., 2008; Schmidt et al., 2013; Arai and Jonas, 2014; Vyleta and Jonas, 2014; Nakamura et al., 2015). A series of recent studies have now begun to link super-resolution imaging with electrophysiology, mathematical modeling, and genetics to explore the physical basis of coupling distances.

The (M)unc13 superfamily of proteins have been recently identified as the release site-generating component (Böhme et al., 2016; Sakamoto et al., 2018). Taking into account that the distance from release sites to VGCCs instruct the efficacy of release, synaptic transmission speed and, ultimately, short-term plasticity, the next eminent step was to determine physical distances between the VGCCs and (M)Unc13 superfamily members (Eggermann et al., 2012; Vyleta and Jonas, 2014).

Recent efforts to obtain visual evidence of VGCC coupling distances to release sites have employed a combination of super-resolution imaging at the *Drosophila* NMJ synapses. Here, STED imaging has revealed a central localization of VGCC alpha1 subunit- Cac, directly under the BRP scaffold (Fouquet et al., 2009). At these synapses, STED resolved images have allowed distance measurements from a VGCC cluster center to the (M)Unc13 cluster position (note that coupling distances in other studies have been measured as the distance between SVs and the respective closest VGCC). Taking advantage of the “neatly arranged” AZs of *Drosophila*, the distances from Unc13A at the presynaptic membrane, to the central localizations of Cac were measured from STED images and electron micrographs of T-bar structures, Both techniques measured Unc13A-VGCC cluster to cluster distances of 50–70 nm (**Figures 2A–G**) (Böhme et al., 2016). In addition, Unc13A mutagenesis experiments show that the N-terminal region of Unc13A mediates release site positioning, with deletions in this region resulting in highly mobile and unspecific positioning of Unc13A across the presynaptic membrane, which, in turn, enabled docking of SVs in aberrant positions. Functionally, this situation resulted in an unusually strong facilitation and loss of temporal precision of SV release (Reddy-Alla et al., 2017).

## Active Zone Assembly

Thus far, we have discussed how release sites are ultrastructurally and molecularly organized at matured synapses. Though it is of crucial importance for the understanding of circuit remodeling during development and learning processes, how in fact do AZs assemble during these processes. We still know relatively little about AZ assembly during development, and this comes mostly from invertebrate preparations. We describe in the following section emerging principles by which AZ scaffolds seem to assemble, and how these assembly processes might intersect with release site formation.

### A Tale of Seeding and Maturation

Analysis of developmental AZ assembly is dominated by the loss-of-function studies in *C. elegans* and *Drosophila* larvae, as these, due to their transparent nature, allow for efficient *in vivo* imaging of their comparatively fast and stereotyped synapse formation program. Rigorous genetic analyses in *C. elegans* has uncovered major regulatory factors controlling presynaptic assembly. Here, Syd-2 and Liprin-α/Syd-2 superfamily members were found to be important for AZ assembly.

Rigorous genetic analyses in *C. elegans* over the years have uncovered major regulatory factors controlling presynaptic assembly. Studies began with the identification of Syd-2, a Liprin-α/Syd-2 superfamily member, important for synapse assembly at *C. elegans* synapses. At *C. elegans* hermaphrodite-specific neuron (HSNL) synapses, Syd-2 gain-of-function studies have shown the protein’s prime role in operating AZ size and shape, thus promoting the formation of new AZ sites, in the absence of Syd-1 (Dai et al., 2006). Following studies on *syd-1* and *syd-2* double mutants have revealed that the Syd-1 and Syd-2 can operate together to promote AZ assembly since at these HSNL synapses a substantial loss in SV and AZ proteins were observed (Dai et al., 2006; Patel et al., 2006). In addition, these studies also revealed that the AZ assembly process is also dependent on the expression of an additional AZ protein: ELKS-1, which is required for Syd-2 function (Dai et al., 2006; Patel et al., 2006). Loss of ELKS-1 by itself, however, did not cause AZ assembly defects, pointing toward a partial redundancy in the ELKS-1 function, since it acts in concert with Syd-1 and other AZ proteins to positively regulate synapse assembly (Dai et al., 2006). Furthermore, elegant genetic interaction experiments between Syd-1 and Syd-2 have identified downstream players regulating the AZ assembly process. At first, both *syd-1* and *syd-2* mutants lose a cohort of synaptic proteins, such as RAB-3, GIT, SAD-1, Unc57/endophilin, and SNN-1/synapsin-1, reaffirming their principal role in recruiting synaptic proteins to the AZ (Patel et al., 2006). Interestingly, these studies also identified a hierarchy between Syd-1, Syd-2 and downstream factors in synapse assembly. Notably, experiments in HSNL synapses show that while Syd-2 overexpression could rescue SV recruitment deficits in the *syd-1* mutants, Syd-1 overexpression did not do so in the *syd-2* mutant background. Thus, Syd-1 evidently acts upstream of Syd-2 in the AZ assembly line (Dai et al., 2006; Patel et al., 2006).

Complete loss of Syd-2 causes AZs to unravel and become elongated, impairing SV docking and synaptic transmission, thus, implicating Syd-2 as an organizer of AZ proteins at the release site (Stigloher et al., 2011; Kittelmann et al., 2013). In line with these findings, Syd-2, similar to its mammalian counterpart Liprin-α, colocalizes with other core AZ proteins ELKS-1, Unc10, and Unc13 at *C. elegans* NMJs, and was found to regulate docking and fusion of SVs at these synapses with these interaction partners (Ackermann et al., 2015). Loss-of-function studies of Unc10 (RIM superfamily member) and Syd-2, for instance, show reduced numbers of docked vesicles at dense projection sites and significantly diminished evoked synaptic transmission (Weimer et al., 2006). In addition, mislocalized SVs were observed docking far away from dense projections in *unc10* mutants, and an overall reduction in the size of dense projections was observed at *syd-2* mutant synapses (Stigloher et al., 2011; Kittelmann et al., 2013). These observations positioned Syd-2 and Unc10 as prime assembly factors for generating AZs (with their release sites) at *C. elegans* synapses.

Further investigations of HSNL synapses revealed the involvement of upstream regulators in the process of AZ assembly. Here, the regulator of synaptogenesis-1 (RSY-1), a synapse disassembly factor, and SYG-1/Neph1 delineate the location of new AZ sites at the HSNL presynaptic terminus. The *rys-1* mutants exhibit excessive synapse formation and RSY-protein directly and physically interacts with Syd-1. Syd-1, Syd-2 and ELKS-1 show a high affinity to each other in cell assays, suggesting they form a complex during AZ assembly. RSY-1, is hence, implicated as a negative regulator of this process and potentially acts by weakening the Syd-1—Syd-2 interaction within the AZ assembly complex, by binding to Syd-1 (Patel et al., 2006; Patel and Shen, 2009). Conversely, the SYG-1/Neph1 proteins seemingly specify the position of the presynaptic assembly process by recruiting Syd-1 and Syd-2. These studies when taken together place Syd-1 and Syd-2 as the central players in the regulation of AZ assembly via interactions with ELKS-1, at SYG-1/Neph1-defined sites or pruning of synapses via interaction with RSY-1, the operant negative regulator, at *C. elegans* synapses.

The *C. elegans* genetic studies (predominantly at HSNL synapses) have beautifully illustrated the hierarchical interplay involved in AZ seeding and assembly. At *Drosophila* NMJ synapses, the temporal sequence of seeding and maturing events at developing AZs could be observed directly using intravital imaging techniques, which capitalize on the transparent nature of the larval cuticle. Super-resolution and intravital fluorescence imaging investigations at the larval NMJ have, thus, shed light on the development of glutamatergic synapses *in vivo*.

Here, assembly of new AZs is mediated by Syd-1/Liprin-α (alias Syd-2) complexes that nucleate the AZ assembly process (Owald et al., 2010). Notably, intravital imaging shows effective accumulation of Liprin-α at the membrane and points of assembly to depend on Syd-1 but not vice versa, a result which corresponds nicely to the genetically retrieved hierarchy worked out at *C. elegans* synapses (Owald et al., 2010).

Conversely, a disassembly of the AZ scaffold has been described at *Drosophila* photoreceptor synapses (Sugie et al., 2015). Chronic exposure to high light levels elicits a disassembly of the AZ scaffold here, which might, at least in part, operate in reverse to the assembly sequence of NMJ AZs (Banovic et al., 2010; Owald et al., 2010, 2012; Sugie et al., 2015). However, not all AZ components disassemble completely during prolonged light exposure, as Syd-1 and VGCC a1 subunit-Cac signals remained rather unaffected in the course of this light triggered disassembly process (Sugie et al., 2015). Liprin-α and RIM-BP were diffusely distributed upon prolonged light exposure as well but did not vanish completely from the AZ, suggesting that they potentially remain in the proximity of the AZ for quick retrieval, as per need, into dynamically changing AZs (Sugie et al., 2015).

Coordinating synapse assembly requires signaling across the synaptic cleft, which separates pre- from postsynaptic membranes. Trans-synaptic pairs of cell adhesion molecules are obvious candidates for coupling AZ and PSD assembly and trigger the AZ assembly of nascent AZs. A complex between presynaptic Nrxs and postsynaptic Nlgs can provide the trans-synaptic signal required to trigger synapse assembly (Scheiffele et al., 2000; Dean et al., 2003; Missler et al., 2003; Li et al., 2007; Zeng et al., 2007; Choi et al., 2011; Owald et al., 2012). How this trans-synaptic signaling axis integrates with the cytoplasmic assembly machinery is, however, less understood.

**FIGURE 3:**
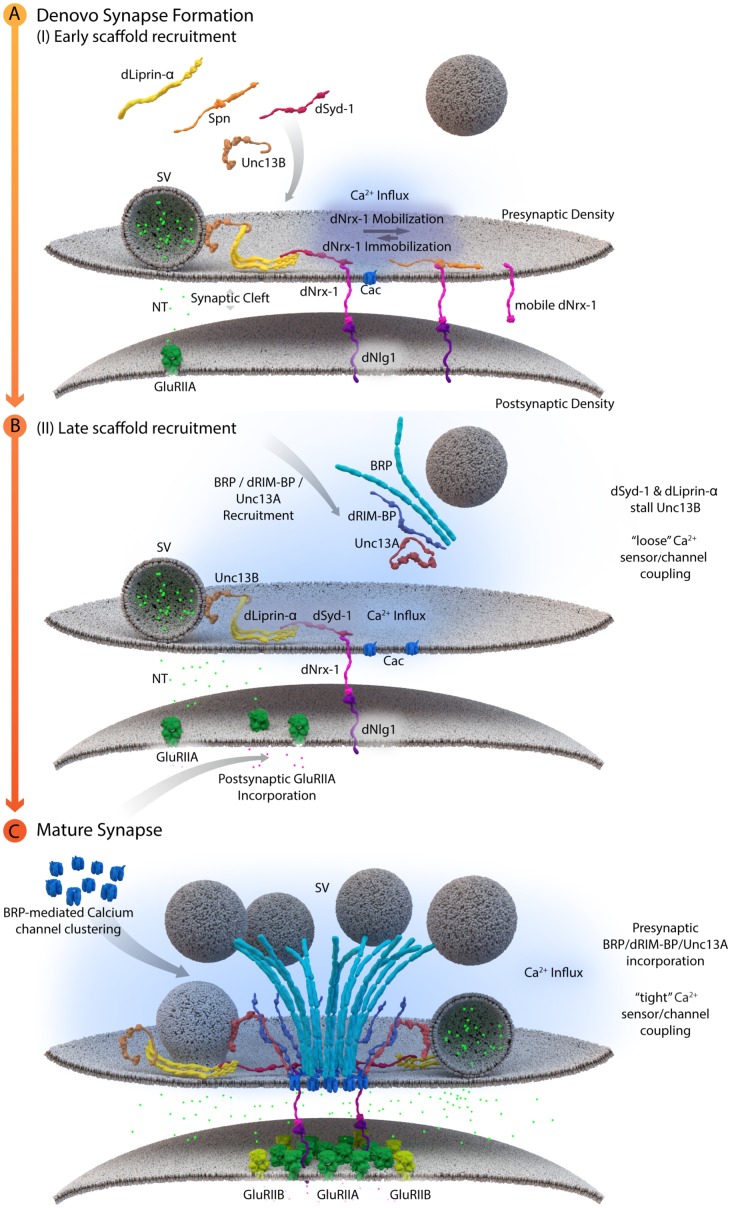
A schematic depicting AZ assembly at *Drosophila* NMJs. **(A)** Depicts a schematic of the early AZ scaffold recruitment and assembly. Here, Spn (Spinophilin) acts antagonistically to dSyd-1 (*Drosophila* Syd-1). Immobile dNrx-1 (*Drosophila* Neurexin-1) molecules get stabilized at the presynaptic membrane by an interaction with the PDZ domain of dSyd-1. Once dNrx-1 is immobilized at the presynaptic membrane, it forms a bridge with its postsynaptic partner dNlg1 (*Drosophila* Neuroligin1). In this way, trans-synaptic contact can also initiate pre- and postsynaptic scaffold assembly. Spn competes with dSyd-1 to bind dNrx-1, with its PDZ domain, thus reducing the amount of dNrx-1 available for dSyd-1 binding. This mechanism helps control the seeding and ultimately the number of AZs at a presynaptic bouton. In addition, dLiprinα also binds to dSyd-1 and together these proteins are known to recruit Unc13B to nascent synaptic sites. Unc13B mediated loose coupling of SVs, facilitates SV-fusion and thus NT (neurotransmitter) release into the synaptic cleft. **(B)** Illustrates a schematic of the late AZ scaffold assembly process. Once a nascent site is established, incorporation of postsynaptic GluRIIA (Glutamate receptor type IIA) on the postsynaptic scaffold is observed, while BRP (Bruchpilot); dRIM-BP (*Drosophila* RIM-binding protein), and Unc13A proteins are recruited in a second wave of AZ scaffold assembly and center themselves at VGCC localizations. **(C)** Illustrates the AZ scaffold maturation process at a developing AZ. A stable AZ scaffold forms with BRP and dRIM-BP centered on the VGCCs; here represented by Cac (*Drosophila* Cacophony). Pre-existing dLiprinα and Unc13B are pushed towards the extremities of the AZ scaffold. The N-terminus of dSyd-1 localizes close to the C-terminal region of BRP. In addition, dSyd-1 still maintains its binding partners the dNrx-1 and dLiprinα, thus tethering the early scaffold to the late AZ scaffold structure. BRP mediated VGCC clustering occurs at the presynaptic terminus, while at the postsynapse the incorporation of GluRIIB (Glutamate receptor type IIB) receptors, predominantly occurs outside the GluRIIA receptor field. Unc13A localizes SVs for tight coupling at approximate distances around 70 nm from the center of the scaffold.

Cytoplasmic AZ assembly factor Syd-1 was found to bind the intracellular C-terminus of *Drosophila* Nrx-1 at *Drosophila* NMJ synapses, thereby promoting the synaptic clustering and immobilization of Nrx-1. As a result, *syd-1* mutants suffer from defective Nlg-1 clustering at postsynaptic sites (Owald et al., 2012). Consistent with the successful formation of this “ménage à trois,” where Syd-1/Nrx-1/Nlg-1 are of prime importance, postsynaptic glutamate receptor incorporation was affected in *syd-1*, *nrx-1* and *nlg-1* single mutants (Owald et al., 2012). In addition, recent work at NMJ synapses identified an additional layer of AZ assembly control. Here, the conserved scaffold protein Spinophilin (Spn) binds to Nrx-1, via its PDZ domain, and thus appears to act in competition with Syd-1. As mentioned above, the current data suggest that Syd-1 protein stabilizes Nrx-1 at newly forming AZs and promotes the overcoming of an “assembly threshold or hurdle” required to execute the assembly toward a defined mature size (Owald et al., 2012). Absence of Syd-1 reduces the number of successful assembly events and at these remaining events, thus AZs appear overgrown as excess amounts of transported cargoes are delivered into these few sites. By contrast, in the absence of Spn, “excessive seeding” seems to take place, which means that too many AZs form, however, they appear to be too small. One explanation might be that Spn binding to Nrx-1 is incompatible with the process of Nrx-1 binding and recruiting Nlg-1. This would consequently lead to an excess of Nrx-1/Syd-1 complexes in *spn* mutants, which triggers new assembly at too many AZ sites, resulting in the distribution of AZ material over too many AZs, and these would, as a result, remain smaller than normal (see **Figure 3A**, Muhammad et al., 2015).

These early and *de novo* presynaptic AZ assembly processes may temporally precede postsynaptic scaffold recruitment by immobilizing Nrx-1 and providing more stable Nrx-1 surfaces for recruitment of the postsynaptic scaffold, via Nlg1 molecules, to places of postsynaptic assembly. However, it appears more likely that aspects of pre- and postsynaptic scaffold recruitment occur in a temporally, tightly coupled fashion and that they reciprocally tune each other continuously. Previous intravital imaging studies at NMJ synapses, showed that the assembly of the postsynaptic specializations is also a temporally protracted process, with the GluRIIA subunit containing glutamate receptor complexes preceding the GluRIIB-containing complexes in the *in vivo* assembly sequence (Rasse et al., 2005; Schmid et al., 2008; Fouquet et al., 2009). As the presynaptic AZ scaffold matures, a visible shift toward DGluRIIB complex incorporation occurs which correlates with increased amounts of BRP in the presynaptic terminus (Schmid et al., 2008) (**Figure 3**). This “parallel scaling” of PSD scaffold proteins with the presynaptic AZ scaffold implicates cross-talk between the pre- and postsynaptic compartments of an AZ, possibly to promote and regulate sequential assembly processes. Bone morphogenetic protein like ligands (Glass bottom boat) secreted by the muscle, which are then sensed by presynaptic receptors (Wishful Thinking), have been implied in such processes (Aberle et al., 2002; Marqués et al., 2002; McCabe et al., 2003; Keshishian and Kim, 2004).

Mechanisms of regulating the early assembly and seeding of AZ scaffolds are also heavily implicated in the dynamic redistribution of AZ material between individual AZ scaffolds. For instance, in general, lower exchange rates of stably incorporated BRP are observed in FRAP experiments between AZs of matured *Drosophila* NMJ synapses, as compared to BRP exchange rates between immature NMJ AZs (Fouquet et al., 2009). Although, within an AZ BRP mediated increase in release probability has been shown to scale with AZ size (Ehmann et al., 2014). Here, the small GTPase Rab3 controls the dynamic protein composition of the release machinery, through its influence over BRP distribution (Graf et al., 2009; Ehmann et al., 2014).

Such early presynaptic mechanisms are fundamental in dictating the correct sequence of AZ seeding, and subsequent spatiotemporally accurate assembly and scaling of the AZ scaffold, thus exerting an influence on the proper localizations of active release sites and the overall activity of an AZ.

### Developmental Maturation of SV–VGCC Coupling During AZ Assembly

As described above (Chapter: Release sites and the control of SV-VGCC coupling), synaptic transmission operates at different VGCC to Ca^2+^ sensor distances, with “loose” or microdomain coupling versus “tight” or nanodomain coupling, in existing regimes (Bucurenciu et al., 2008; Schmidt et al., 2013; Vyleta and Jonas, 2014; Wang and Augustine, 2015). Of note are the beautiful biophysical analyses that finds coupling distances to undergo developmental tightening, expressed in changes of release probabilities and short-term plasticity at the Calyx of Held and cortical mammalian synapses (Fedchyshyn and Wang, 2005; Baur et al., 2015). In conjunction, quantitative models have linked coupling distances and VGCCs numbers. This model was built off of data from immature calyx synapses, exhibiting microdomain coupling, which presented 5-6 VGCCs, localized at ∼60 nm from an SV, that were engaged in a single SV fusion event. Once matured and coupled into nanodomains, at the same synapses, only 2–3 VGCCs, positioned ∼20 nm from an SV, were estimated to be involved in a single SV fusion event (Wang et al., 2009). In addition, nanodomain coupling at mature calyxes exhibits a rise in Ca^2+^ concentrations, which reach Ca^2+^ sensors, from 35 to 56 μM and, hand in hand, a rise is seen in SV release rates from ∼600 SVs/ms to 1000 SVs/ms at these AZs (Wang et al., 2008). During developmental AZ maturation, such factors and mechanisms of developmental tightening may potentiate the differential spacing of the SVs and VGCCs to defined distances on the presynaptic membrane. However, the molecular mechanisms behind this tightening process have not, as yet, been worked out at the Calyx of Held.

**FIGURE 4:**
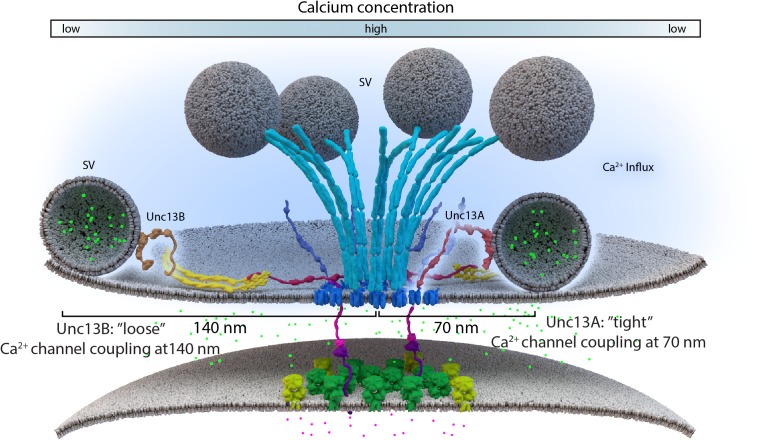
Loose and tight coupling at *Drosophila* NMJ AZs. A schematic of loose and tight coupling occurring at single NMJ AZs. Loose coupling of SVs, depicted on the left-hand side of the AZ, occurs at low Ca^2+^ concentrations, at a rough distance of 140 nm from the AZ center and localize at Unc13B signals that are positioned 120 nm from the center. Tight coupling, depicted on the right-hand side of the AZ, occurs near high Ca^2+^ concentrations, that is typically positioned at a 70 nm distance from the AZ center or at Unc13A localizations, which are roughly positioned 50 nm from the AZ center.

Lack of the filamentous protein Septin5 at Calyx of Held synapses reportedly transforms AZ coupling precociously from an immature microdomain coupled into more matured nanodomain coupling type. Septin5 might establish a physical barrier precluding SV docking/priming at positions too close to VGCCs in the immature state (Yang et al., 2010). However, controlling the relative exact spacing of SVs and VGCCs during developmental and activity-dependent tightening might well require additional proteins, which are likely other AZ scaffold proteins. (M)Unc13 superfamily proteins are prime molecular candidates to link release site definition to spacing control since they interact with both AZ scaffold factors and SV proteins, (Acuna et al., 2015; Böhme et al., 2016; Wang et al., 2016a; Sakamoto et al., 2018). Isoforms of this family have been shown to not only define release sites locations, at *Drosophila* NMJ synapses, but also establish differential coupling distances via their distinct N-termini (Böhme et al., 2016; Reddy-Alla et al., 2017). A combination of super-resolution light and EM studies have shown that Unc13A is positioned to dock SVs about ∼50–70 nm from the VGCC cluster center in the middle of the AZ (Böhme et al., 2016). In the same system, with easy accessibility to intravital live imaging, maturation of coupling distances was recently investigated by imaging Unc13 isoforms together with early and late scaffold complex proteins Liprin-α/Syd-1 and BRP/RIM-BP, respectively, during the assembly of NMJ AZs. In this study, Unc13B colocalizes with the “early” Liprin-α/Syd-1 scaffold proteins and even its accumulation at AZs sites was found to rely explicitly on the presence of these early scaffold proteins. Consequently, at matured AZs, Unc13B appears clustered at greater distances from VGCCs (>100 nm), along with the other early AZ players. Conversely, Unc13A was positioned and stabilized in discrete clusters via the BRP-RBP scaffold close to presynaptic VGCCs (<100 nm), during the later stages of the assembly process (**Figure 3C**) (Böhme et al., 2016). This mechanism integrates a sequence of (M)Unc13 superfamily members into the assembly and maturation of release sites and overall into AZ development. During maturation, the late AZ scaffold incorporates Unc13A near VGCCs; it is hence the Unc13A isoform at mature CAZs that tightens the spatial coupling of release-ready SVs to presynaptic VGCCs and subsequently tunes NT release (Fouquet et al., 2009; Liu et al., 2011; Böhme et al., 2016). These results point to the parallel existence of two functional exocytosis pathways, probably with similar Ca^2+^ sensing and fusion mechanisms, regulated by the different Unc13 isoforms. The exact spatiotemporal and differential positioning of the two Unc13 isoforms, within the release sites, thus ultimately influences the maturation and function of AZ scaffolds (Böhme et al., 2016) (**Figure 4**).

In addition, a new study at *Drosophila* brain synapses shows that the “coupling rules” here are similar as to those found at NMJ synapses (Böhme et al., 2016). Here again, BRP and Syd-1 mediate control of Unc13A and B accumulations, respectively. Loss of either Unc13A or B did not, however, affect BRP or Syd-1 distributions, which implies that these specific scaffold components recruit and position the Unc13 isoforms, not vice versa (Fulterer et al., 2018). Detailed analysis of synapse types of the olfactory circuits of the *Drosophila* brain have revealed high levels of BRP/Unc13A at their first relay synapses, which, in turn, fostered high release probabilities at these AZs and thus, support the fast but depressing release at these synapses. By comparison, the second relay projection neurons and the interneuron synapses presented high Syd-1/Unc13B levels that consequently promoted “loose” coupling and lower release probabilities at these synapses (Fulterer et al., 2018). This mechanism argues in favor of the presence of tight and loose coupling via (M)Unc13 scaffolds as a design feature to tune and diversify release features across synapses, to adapt them to their specific needs within the relevant circuits.

It will be interesting to see whether a similar logic applies to mammalian synapses as well. Notably, bMunc13-2, a brain-specific mammalian (M)Unc13 superfamily member that also lacks the C2A domain, is recruited by ELKS (homologous to BRP) to AZ sites (Kawabe et al., 2017). Work on mammalian synapses eludes toward similar trends for coordinating synaptic release. Studies at Calyx of Held synapses demonstrate the “Exclusion Zone” principle, wherein a minimal distance between VGCCs and SVs, in which no SVs can fuse, allow for proper control of release sites (Keller et al., 2015). In a pioneering study, the distinct spatial distribution of Munc13 isoforms at the calyx of Held synapses show Munc13-1 localized to the center of release sites and Munc13-2/3 to peripheral release sites, where Munc13-2 and Munc13-3 together control the slow SV pool contribution to the RRP rather than the fast SV pool and, in concert with Munc13-1, regulate release probability and the RRP of SVs at these synapses (Chen et al., 2013). Preferentially loose “microdomain” coupling was observed at immature AZ sites at these same synapses, while a tight “nanodomain” coupling was more readily observed at mature AZ sites (Wang et al., 2008; Chen et al., 2015; Nakamura et al., 2015). In addition, a recent study on parallel fiber AZs showed that Munc13-3 mediates the regulation of nanodomain coupling at these AZs and regulates Cav2.2 (N-type) and Cav2.1 (P/Q-type) channel localizations differentially to these AZs (Kusch et al., 2018). These insights reinforce the role of the (M)Unc13 family of proteins, at mammalian CNS and *Drosophila* NMJ synapses, in defining release sites and the developmental regulation of coupling distances.

Furthermore, work at the *C. elegans* synapses also displays a differential expression of its two Unc13 isoforms. Unc13L regulates fast release, here, while slow release requires both Unc13L and Unc13S isoforms. Close spacing of the Unc13L isoform to VGCCs was necessary for accelerating NT release and maintaining fast, evoked release at high release probabilities. In an analogous fashion, slow SV release was found to localize mostly outside presynaptic AZ sites (Hu et al., 2013; Zhou et al., 2013).

Taken together, work from mammalian, *Drosophila*, and *C. elegans* AZs suggest that (M)Unc13 isoforms follow a sequential developmental assembly sequence, with apparently loose coupling preceding late coupling during AZ maturation, that is mediated through the differential arrival of AZ scaffold protein complexes. This points toward the presence of a fundamentally conserved mode of tuning synapse release features, across synapse types, that is regulated by the (M)Unc13 superfamily members.

## Conclusion and Future Directions

Decades of work have revealed a conserved subset of AZ scaffold proteins that collectively organize AZs functionally and structurally, and whose relative amounts and isoform spectrum might well be a major means of synapse diversification. Extensive loss-of-function studies of subsets of these core member proteins have revealed at least partial redundancies between AZ scaffold components, and have also provided a fuller appreciation of the multiple, sequential steps within the processes of AZ scaffold assembly and maturation. The AZ scaffold assembly process is based on defined and dynamically regulated protein-protein interactions that employ a conserved set of interaction surfaces including both intra- and intermolecular coiled-coil interactions, as well as SAM and PDZ domain interactions (Südhof, 2012). Bearing in mind that more than just nuanced differences occur at varying synapses to bolster the specific functional needs of individual synapses types, these mechanisms ultimately converge to coordinately regulate release site generation and function within any given AZ.

Research is now at the precipice of understanding how AZ scaffolds determine the generation of a new release site and the coordinated sequence of events that promote SV fusion. It could be ascertained that Munc13-1 and Unc13A, both members of the (M)Unc13 family, are the dominant release site-generating molecules (Reddy-Alla et al., 2017; Sakamoto et al., 2018). In addition, the propensity of (M)Unc13 isoforms to differentially localize at AZ sites of *Drosophila, C. elegans*, and mammalian synapses, to regulate the effective “tight” and “loose” SV coupling to VGCCs, provides a first insight into the developmental and regulatory release site mechanisms on AZ function. (M)Unc13 superfamily members likely exhibit similar functions at different synapses as a result of an evolutionarily conserved design, which may be in place, as a means to adapt to various types of activating stimuli and optimize the speed and reliability of synaptic transmission. Although, the temporal developmental maturation of a release site may be the first big step toward understanding release site modulation on AZ function, how release site specifications regulate short- and long-term, activity-dependent, plasticity warrants future investigations.

A closer look at the coupling regulated by the (M)Unc13 release site-generating molecules reveals the presence of two scaffold proteins, Syd-1 and BRP, at its core that potentiate loose and tight coupling through (M)Unc13 members at a variety of synapse types in *Drosophila* (Fulterer et al., 2018). Thus, it would be of imminent interest to discern whether Syd-1 and ELKS/Cast/BRP superfamily members regulated coupling might be a conserved feature in synapses of other model systems. The AZ scaffold-regulated Unc13A and B levels have also been shown to provide a means to vary and tune synaptic release to support functional diversity (Fulterer et al., 2018). Perhaps it would also be of prudent interest to delve further into the relationships between the AZ scaffold and postsynaptic and/or trans-synaptic factors, such as cell adhesion molecules, for example, tenuerins, LRP4 and Elfn1, which may be involved in regulating synaptic plasticity (Hong et al., 2012; Sylwestrak and Ghosh, 2012; Mosca et al., 2017)

In light of these recent advances, the AZ scaffold may very well promote generation, localization, and stabilization of release site slots by exerting influence upon the (M)Unc13 superfamily of proteins and their molecular levels as an evolutionary means to diversify synapses (Fulterer et al., 2018; Nusser, 2018).

The *Drosophila* NMJ synapses have been particularly successful in applying a combination of genetic modulation with super-resolution microscopy and intravital live imaging. With this powerful blend of techniques, recent work at these synapses has been particularly instructive in providing the temporal details of AZ scaffold assembly and maturation, building upon work from C. elegans, while also providing nanoscopic details of release site coupling that corroborate observations from mammalian synapses. This potent mix of imaging techniques and favorable genetic accessibility available for *Drosophila* NMJ investigations places a bright future for work in this model system to address a multitude of salient questions emerging now in the subject of release site regulation.

## Author Contributions

TG and SS both contributed equally to the writing and editing of this piece.

## Conflict of Interest Statement

The authors declare that the research was conducted in the absence of any commercial or financial relationships that could be construed as a potential conflict of interest.

## Abbreviations

AZ: active zone
BRP: Bruchpilot
Ca^2+^: calcium
Cac: cacophony
CNS: central nervous system
EM: electron microscopy
ELKS: glutamic acid (E), leucine (L), lysine (K) and serine (S)-rich protein
FRAP: fluorescence recovery after photobleaching
GluRIIA/B: glutamate receptor type IIA/B
HSNL synapses: C. elegans hermaphrodite-specific neuron synapses
LAR: leukocyte common antigen-related
Nlg: neuroligin
NMJ: neuromuscular junction
NT: neurotransmitter
Nrx: neurexin
RIM: Rab3-interacting molecule
RIM-BP: RIM-binding protein
RRP: readily releasable pool
SNARE: soluble NSF Attachment Protein REceptor
Spn: spinophillin
SVs: synaptic vesicles
Syd-1: synapse defective-1
VGCCs: voltage-gated Ca^2+^ channels.
